# Association Between Self-Reported Health and Education with Past-year Dental Visits among Older Mexican Adults: Results of the Mexican Health and Aging Study 2018

**DOI:** 10.3290/j.ohpd.b3500629

**Published:** 2022-10-19

**Authors:** Alvaro García Pérez, Alvaro Edgar González-Aragón Pineda, Jacqueline Adelina Rodríguez Chávez, Teresa Villanueva Gutiérrez, Nora Guillermina Pérez Pérez

**Affiliations:** a Professor, Laboratory of Public Health Research, Faculty of Higher Studies (FES), Iztacala, National Autonomous University of Mexico (UNAM), Mexico City, Mexico. Designed the study, contributed to analyzing the data, wrote and reviewed the manuscript, contributed to the discussion.; b Professor, Laboratory of Public Health Research, Faculty of Higher Studies (FES), Iztacala, National Autonomous University of Mexico (UNAM), Mexico City, Mexico. Contributed to analyzing the data, reviewed the manuscript, contributed to the discussion.; c Professor, Department of Comprehensive Dental Clinics, University Center for Health Sciences, University of Guadalajara, Guadalajara, Mexico. Reviewed the manuscript, contributed to the discussion.; d Professor, Health Care Department, Metropolitan Autonomous University-Xochimilco, Mexico City, Mexico. Reviewed the manuscript, contributed to the discussion.; e Professor, Laboratory of Public Health Research, Faculty of Higher Studies (FES), Iztacala, National Autonomous University of Mexico (UNAM), Mexico City, Mexico. Reviewed the manuscript, contributed to the discussion.

**Keywords:** educational status, multimorbidity, older adults, past-year dental visits, self-report

## Abstract

**Purpose:**

To explore the relationship between of self-reported health (SRH) and educational attainment with past-year dental visits in older adults in México.

**Materials and Methods:**

For this cross-sectional study, data were derived from the Mexican Health and Aging Study 2018 (MHAS-2018), which used a nationally-representative sample of older adults (50 years or older) in Mexico (n = 14,085). Variables taken from the MHAS questionnaire included residence (rural/urban), years of education, SRH, multimorbidity, pain severity, and past-year dental visits. A logistic regression model was used to identify the association between the variables and past-year dental visits.

**Results:**

While the percentage of past-year dental visits reported was 39.8%, this number declined with age [OR=0.76, p < 0.001], with older adults living in rural areas 34% less likely to report past-year dental visits than older adults living in urban areas. Older adults with no formal education were 73% less likely (OR=0.27; p < 0.001) to report past-year dental visits than older adults ≥10 years education. Older adults with poor SRH were 32% less likely (OR=0.68; p < 0.001) to report past-year dental visits than older adults with good/very good/excellent SRH. Sex, degree of multimorbidity, and pain level ([OR=1.37; p < 0.001] [OR=1.37; p < 0.001] and [OR=1.17; p < 0.001]) were all found to be positively associated with past-year dental visits in the study population.

**Conclusion:**

The present study highlights the association between poor SRH, a low level of educational attainment, and less frequent past-year dental visits, as well as the finding that past-year dental visits declined with age in the older adults sampled.

The analysis of health status and healthcare is used to demonstrate post-implementation changes and progress of a healthcare treatment program or intervention in different populations. Changes in healthcare result from complex interactions between the demographic characteristics and cultural, economic, psychological, social, and environmental factors, and the evaluation thereof may help plan health policy.^9,33^

SRH is a qualitative health indicator, which is easy to use via a subjective question: How would you characterize your general health? The response options range from poor to excellent. The use of this question may be of great significance, as it can serve as an indicator of a participant’s real state of health.^33^

SRH has been used in various epidemiological surveys in older adults, due to the fact that it is an indicator associated with mortality and a person’s general welfare.^34^ It is also used as predictor of mortality, given the tendency of older adults to consistently provide information pertaining to their clinical and subclinical conditions.^4^ It has also been shown that SRH is a stable variable in the majority of the population and different age groups,^13^ while its use indicates perceived well-being and may highlight health inequalities present in the population.

An association between SRH and chronic degenerative diseases, such as cardiovascular conditions,^8^ cancer, chronic respiratory disease (such as chronic obstructive pulmonary disease and asthma),^18^ and diabetes,^1^ has been found, while a relationship between SRH and oral health problems in older adults has also been documented.^4,16^

An ever-closer relationship has been identified between SRH and both economic status/income and education in different population groups. Educational level is one of the most commonly used indicators in epidemiological studies evaluating health inequalities, as those with a low socioeconomic status and a low level of education are more likely to be in poor health.^29^ Even in developed countries, it has been observed that adults with less education present poorer health than other demographic groups.^36^

Oral health problems in older adults are of great importance, since at this stage of life they could have a negative impact on quality of life,^21^ and poor oral health may have consequences for general health as well. During aging, different conditions can occur that affect the oral cavity, such as tooth loss, caries, and periodontitis.^11^ Worldwide, caries and periodontal disease are the leading causes of tooth loss in older adults (≥ 60 years).^14^ Despite the importance of regular visits to dentist and extant treatment need, data from surveys^28^ show that older adults use dental services less frequently.^3^

Little evidence has been obtained, however, in Latin America on the relationship between SRH, level of educational attainment, and the frequency of dental visits in older adults. Only 46% of the older adults in a population sampled in the United States were found to visit the dentist for a general examination.^26^ Nitschke et al^20^ showed that dental service utilization is negatively associated with age. The additional influencing factors on dental service utilization included educational level, pain, and financial situation.^20^

Those older adults who regularly visit the dentist have been found to enjoy a positive impact in terms of the prevention of oral disease. It has also been observed that the prevention of oral disease is directly related to short intervals between dental visits, while long intervals delay the diagnosis, prevention, and treatment of oral disease.^31^ Also, education is thought to influence the way individuals achieve and maintain oral health through the perception of the need for dental services.^3^ Accordingly, it was hypothesized that the use of past-year dental visits is associated with SRH and educational attainment, independent of other socioeconomic characteristics. Therefore, the present study aimed to explore the relationship between of SRH and educational attainment with past-year dental visits in older adults in México.

## Materials and Methods

### Data and Ethical Approval

The MHAS is the first nationally-representative longitudinal study of aging in Mexico and has been ongoing since 2001, with corresponding interviews and data collection carried out in people over 50 years old from urban and rural areas of the 32 states that comprise the Mexican Republic. The questionnaire collects information pertaining to demographic data, general health status, chronic conditions, socioeconomic status, migration, family structure, and housing conditions. The MHAS surveys are conducted under the supervision of coordinators from both Mexico and the United States and are partially funded by the National Institute of Aging at the United States’ National Institutes of Health (NIH R01AG018016) and Mexico’s Instituto Nacional de Estadística y Geografía (INEGI or National Institute of Statistics and Geography). The MHAS data files and documentation are available for public use at www.ENASEM.org.^17^ Research carried out using the MHAS databases must adhere to the relevant ethical conditions for research on human beings and must be approved by the corresponding ethics committee, i.e. that of the University of Texas Medical Branch and, in Mexico, that of INEGI and the National Institute of Public Health. All participants are required to provide their signed informed consent.

### Study Population

The sample used by the present study was taken from the MHAS-2018 survey and comprised 14,085 adults aged 50 years and older, of whom 1969 were excluded due to missing responses.

### Independent Variables

The present study analyzed various sociodemographic and health-related covariables, as follows: age (in years), which was divided into two groups (50–64 years and ≥65 years); sex (female/male); multimorbidity in terms of the number of chronic diseases (diabetes, hypertension, heart attack, asthma, and arthritis) with which they had been diagnosed; pain (no pain/mild/moderate/severe); and place of residence (urban/rural). A rural area in Mexico is classified as one with a population of less than 2500 inhabitants.^24^ The present study categorized the variable of educational attainment into no education/1–9 years/≥10 years.

### SRH

The SRH data used by the present study was obtained by means of a standardized questionnaire, which comprised the following questions: “Would you say your health is excellent/very good/good/fair/poor?”; “Comparing your health now with your health two years ago, would you say your health is now… much better/somewhat better/more or less the same/somewhat worse/much worse?”; and, “Compared with other people your age, would you say that currently your health is…better/more or less the same/worse?”

### Outcome Variable: Past-year Dental Visits

The older adults were classified according to their responses to the following question: In the last year, how often have you seen a dentist? They were then categorized into two groups: no visits; and one or more visits.

### Statistical Analysis

Using Pearson’s Χ^2^ test, bivariate analysis was performed on past-year dental visits by age group, sex, educational level, multimorbidity, pain, residence (rural/urban), and SRH. Logistic regression models were performed to ascertain the association between the dependent variable, past-year dental visits (no [0] or one or more visits [1]), and the independent variables (age group, sex, SRH, educational level, multimorbidity, pain, and residence) adjusted for confounders, while the odds ratio (OR) was calculated to a 95% confidence interval (95% CI). The variables for which a result of p < 0.25 was obtained or those that were supported by the theory were used for the construction of the models. Model diagnostic tests were conducted using the Hosmer-Lemeshow goodness-of-fit test, while the analysis was performed using the Stata 15 program (Stata; College Station, TX, USA).

## Results

### Population Characteristics

Of the total number of participants, 57.1% were women (8403) and 49.2% (6042) were men, wiht a mean age of 64.7 (±10.4) years. Furthermore, 80.8% (11378) of participants had ≤ 9 years of education and 19.9% (2796) lived in a rural area. With regard to health characteristics, 60.1% of the participants reported at least one chronic disease, while 25.7% had two or more, of which diabetes (25.2%), hypertension (44.9%), and arthritis (12.5%) were the most frequent. In terms of SRH, 28.9% considered their health good, 53.9% fair, and 9.7% poor. Compared with other people, 46.9% consider that their health is currently more or less the same and 9.8% worse. Additionally, 39.8% of the participants reported frequent pain, the severity of which they described as mild (13.3%), moderate (17.2%), a or severe (9.3%).

Of the total sample, the percentage of past-year dental visits was 39.8% (5609), while the female respondents reported a higher percentage of past-year dental visits than did males (61.3% vs 38.7%; p < 0.001). Adults <65 years reported a higher percentage of past-year dental visits than adults ≥65 years (58.3% vs 41.7%; p < 0.001), while those older adults with a higher level of education reported more frequent past-year dental visits than those with no education (28.6% vs 8.4%; p < 0.001). Older adults in rural areas reported a lower percentage of past-year dental visits than did adults in urban areas (14.0% vs 86.0%; p < 0.001). The results for the sample population are presented in Table 1.

**Table 1:** Sociodemographic and health variables by past-year dental visits among Mexican older adults in Mexico (n = 14,085)

	Past-year dental visits		p*
	None (n = 8476) n (%)	≥ 1 (n = 5609) n (%)	
**Age in years**			
50–64	4064 (47.9)	3273 (58.3)	<0.001
≥ 65	4412 (52.1)	2336 (41.7)	
**Sex**			
Men	3873 (45.7)	2169 (38.7)	<0.001
Women	4603 (54.3)	3440 (61.3)	
**Education (years)**			
No education	1396 (16.5)	472 (8.4)	<0.001
1–9 years	5977 (70.5)	3533 (63.0)	
≥10 years	1103 (13.0)	1604 (28.6)	
**Multimorbidity**			
0	3535 (41.7)	2088 (37.2)	<0.001
1	2797 (33.0)	2039 (36.3)	
2	1653 (19.5)	1170 (20.9)	
≥3	491 (5.8)	312 (5.6)	
**Pain**			
No pain	5171 (61.0)	3314 (59.1)	0.030
Mild	1127 (13.3)	743 (13.2)	
Moderate-Severe	2178 (25.7)	1552 (27.7)	
**Place of residence**			
Urban	6465 (76.3)	4824 (86.0)	<0.001
Rural	2011 (23.7)	785 (14.0)	

Table 2 shows the associations between SRH questions and past-year dental visits (none/one or more), which reveal statistically significant differences among the answers to SRH questions. Older adults with poor SRH presented a lower number of past-year dental visits than adults with good SRH (7.9% vs 31.0%; p < 0.001).

**Table 2:** Associations between self-reported health (SRH) questions by past-year dental visits in older Mexican adults (n = 14,085)

	Past-year dental visits		p*
	None (n = 8579) n (%)	≥ 1 (n = 5651) n (%)	
**Self-reported health** Would you say your health is…			
Excellent/very good	541 (6.4)	513 (9.2)	<0.001
Good	2335 (27.5)	1739 (31.0)	
Fair	4678 (55.2)	2913 (51.9)	
Poor	922 (10.9)	444 (7.9)	
Comparing your health now with your health two years ago, would you say your health now is…?			
Much better	308 (3.6)	238 (4.2)	<0.001
Somewhat better	864 (10.2)	576 (10.3)	
More or less the same	4725 (55.8)	3279 (58.5)	
Somewhat worse	2180 (25.7)	1360 (24.2)	
Much worse	399 (4.7)	156 (2.8)	
Compared with other people your age, would you say that currently your health is…			
Better	3432 (40.5)	2667 (47.6)	<0.001
More or less the same	4078 (48.1)	2524 (45.0)	
Worse	966 (11.4)	418 (7.4)	

### Multivariable Analysis

The results of the logistic regression analysis (Table 3) show that past-year dental visits are positively associated with sex, multimorbidity, and pain severity and are negatively associated with educational attainment, SRH, age, and residence (rural/urban). The results obtained show that the female respondents were 37% (OR=1.37 [1.27–1.47]; p < 0.001) more likely to have visited the dentist in the past year than men. Older adults with moderate/severe pain were 17% (OR=1.17 [1.07–1.28]; p < 0.001) more likely to have visited the dentist in the past year than older adults with no pain.

**Table 3:** Adjusted odds ratios from the logistic regression model for the associations between past-year dental visits and related variables in older adults in Mexico (n = 14,085)

Variables	Crude OR (95%CI)*	p	Adjusted OR (95%CI)*	p
**Sex (male ref.)**				
Female	1.33 (1.24 – 1.42)	<0.001	1.37 (1.27 – 1.47)	<0.001
**Age categories (50–64 years ref.)**				
≥65 years	0.65 (0.61 – 0.70)	<0.001	0.76 (0.70 – 0.81)	<0.001
**Residence (urban ref.)**				
Rural	0.52 (0.47 – 0.57)	<0.001	0.66 (0.60 – 0.72)	<0.001
**Education years (≥10 years ref.)**				
1-9 years	0.40 (0.37 – 0.44)	<0.001	0.43 (0.39 – 0.48)	<0.001
No education	0.23 (0.20 – 0.26)	<0.001	0.27 (0.24 – 0.32)	<0.001
**Multimorbidity (no diseases ref.)**				
1	1.23 (1.14 – 1.33)	<0.001	1.33 (1.22 – 1.44)	<0.001
2	1.19 (1.09 – 1.31)	<0.001	1.37 (1.24 – 1.52)	<0.001
≥3	1.07 (0.92 – 1.25)	0.346	1.26 (1.07 – 1.48)	0.006
**Pain severity (no pain ref.)**				
Mild	1.02 (0.92 – 1.13)	0.588	1.07 (0.96 – 1.19)	0.185
Moderate/Severe	1.11 (1.02 – 1.20)	0.008	1.17 (1.07 – 1.28)	<0.001
Self-Reported Health (Excellent/Very good/good ref.)				
Fair	0.79 (0.74 – 0.85)	<0.001	0.88 (0.81 – 0.96)	0.003
Poor	0.61 (0.54 – 0.69)	<0.001	0.68 (0.59 – 0.78)	<0.001

*OR: odds ratio; CI: confidence interval; p: significance level. Log likelihood: -8997.0791; Hosmer-Lemeshow: 0.3797.

Older adults living in rural areas were 34% less likely to have visited the dentist in the past year than older adults living in urban areas, while older adults with no formal education were 73% less likely (OR=0.27 [0.24–0.32]; p < 0.001) to have visited the dentist in the past year than older adults with ≥10 years of formal education. Finally, older adults with poor SRH were 32% less likely (OR=0.68 [0.59–0.78]; p < 0.001) to have visited the dentist in the past year than older adults with good/very good/excellent SRH.

## Discussion

The present cross-sectional study, using data taken from a representative sample at a national level for older adults in Mexico, found that only 39.8% of the participants had visited the dentist in the past year. Andrade et al^3^ reported a 31.7% prevalence of the use of dental services. Of the covariables used for logistic regression, it was observed that a poor level of SRH, a low level of educational attainment, living in a rural area, and higher age were statistically associated with a low frequency of visits to the dentist in the past year, while one or more comorbidities, being female, and being in pain increased the likelihood of dental visits.

One of the findings of the present study was that, as the age of the respondents increased (≥ 65 years), the probability of visiting the dentist in the past year decreased. In fact, longitudinal research has found that older adults use dental services less frequently than younger adults,^28^ a finding that the present study sought to expand upon, finding that the probability of visiting the dentist decreased as age increased.

SRH is an indicator used to ascertain the general state of health and is a good predictor of adverse events in older adults.^35^ The present study found that older adults with regular or poor self-reported health were less likely (12% and 32%, respectively) to have visited the dentist in the past year. Previous epidemiological studies have shown that 46% of older adults in the US^26^ have visited the dentist in the past year, but in China only 19% did so.^12^ Poor self-rated health is associated with poorer health outcomes, greater disease severity, and symptom burden. The poor oral health – including tooth loss – can decrease function and cause discomfort, consequently possibly affecting self-perceived general health.^30^ Therefore, the use of self-reported health can allow professionals to understand and improve important factors influencing the quality of life of older adults, with the aim of promoting and improving health and reducing inequalities in access to health care/health services in vulnerable populations.

Attaining a certain level of education in their youth can enable older adults to adapt to new conditions. Education is considered an aspect fundamental to both delaying the effects of aging and helping older adults to maintain their well-being.^10^ Similarly, education helps to promote better health by increasing cognitive abilities, thus better enabling people to maintain good health.^5^ On the other hand, it has been reported that adults with a lower educational level present poorer health and use dental services less frequently compared to other populations.^7,22,36^ The present study revealed that older adults with less education present a lower likelihood to have visited the dentist in the past year. The educational attainment of older adults may affect their access to and use of dental services through contributory sociocultural factors and health-related beliefs and attitudes.^3^ Consequently, dental services need to remove education-based barriers to access, not only to reducing the great inequalities in oral health but also to make the services responsive to the population, improving the general well-being of older adults.

It is estimated that 21% of the Mexican population lives in rural areas,^19^ while older adults in rural areas are often geographically isolated and economically and socially disadvantaged, causing difficulties in accessing and affording healthcare services which, in turn, leads to untreated disease.^27^ Likewise, it has been observed that older adults living in rural areas have been identified as a highly disadvantaged population subgroup in terms of access to dental services.^2^ In the US, it has been found that older rural adults are 46.9% less likely to have visited the dentist in the past year.^32^ Older rural adults in Australia rural were 14% less likely to have seen a dentist and remote residents were 27% less likely,^2^ while the present study found that older rural adults were 34% less likely to have visited a dentis in the past year. The use of dental services is influenced by individual factors, lack of access, characteristics of the healthcare system, and the experience of using the services. Some of these factors may be related to the location (rural/urban) of such services.^23^ Given the foregoing, it is of the utmost importance that strategies oriented around the strengthening of primary healthcare services are implemented, given that older adults in rural areas require continual and timely access to healthcare. Moreover, healthcare professionals play a fundamental role in supporting older people, providing high-quality and integrated care.

Multimorbidity is a frequent problem in older adults, in part due to an increasingly large elderly population thanks to the decrease in mortality rates. Scientific evidence has shown that it is also associated with other factors, such as the presence of pain, disability, functional status, quality of life, oral disease, and increasing age.^6,11,25^ The present study found that the presence of two or more comorbidities and moderate/severe pain increases the probability of visiting the dentist in older adults. Regular dental visits are necessary for diagnostic and timely treatment of oral diseases, e.g. oral cancers, as adults over the age of 65 account for 60% of oral cancer-related deaths, despite an 80% cure rate for early diagnosis.^15^ On the other hand, the lack of dental care can compromise an individual’s health status and worsen without treatment. In addition, poor oral health can also exacerbate chronic health conditions, such as diabetes, and affect nutrition caused by tooth loss, which is essential for the physical and mental health/well-being of older adults.^7^ Therefore, our results could indicate that older adults with the presence of multimorbidity have a greater need for dental treatment to improve their oral health.

### Limitations

This study has some limitations. First, the findings are based on cross-sectional data, which do not allow causal inference. Second, given that self-reported health worsens with advancing age, it is probable that countries with a higher proportion of older adults present a lower number who report themselves to be in good health. On the other hand, one of the advantages of the present study is that the sample was representative, for both urban and rural areas, of adults 50 years or older in Mexico. This made it possible to evaluate the impact of chronic disease and aging-related factors on the use of dental services by the population.

## Conclusions

The present study found that 39.8% of the participating older adults had visited the dentist in the past year. The frequency of dental visits in older adults is determined by various factors, such as age, sex, SRH, educational attainment, place of residence (rural or urban), level of multimorbidity, and severity of pain. Aging, along with all the factors related to it, can be seen as a factor increasing the risk of a low frequency of dental visits. It is important to strengthen healthcare strategies that provide older adults with opportunities to improve their general state of health.
